# Techniques for Imaging Vascular Supply of Peripheral Nerves

**DOI:** 10.1055/s-0041-1731280

**Published:** 2021-07-23

**Authors:** Alec Giron, Cameron Cox, Brendan MacKay

**Affiliations:** 1 Department of Orthopaedic Surgery, Texas Tech University Health Sciences Center School of Medicine Lubbock, Texas Tech University Health Sciences Center School of Medicine, Lubbock, Texas, United Sates; 2 Department of Orthopaedic Surgery, Texas Tech University Health Sciences Center, Lubbock, Texas, United States; 3 Department of Orthopaedic Surgery, Texas Tech Health Sciences Center, Lubbock, Texas, United Sates

**Keywords:** imaging, peripheral nerve repair, CT imaging, ultrasound, peripheral vasculature, MRI, CEMRI, surgery

## Abstract

Few studies have been developed to map the vascular structures feeding peripheral nerves, with the majority using cadaveric models and inadequate sample sizes. Preliminary evidence, while limited, indicates that the mapping of these vessels may allow or preclude certain procedures in nerve reconstruction due to the location of essential arterial inflow to the vasa nervorum. This review evaluates the evidence regarding historical, current, and emerging techniques for visualizing these vascular structures in vivo and considers their potential application in peripheral nerve vasculature.

## Introduction

While the exact mechanism is still unknown, it has been shown that vascular supply to peripheral nerves is crucial to both normal function and regeneration after injury. When repairing peripheral nerves, surgeons aim to preserve intact anatomy and avoid mobilizing a nerve in a way that separates it from perforating vessels when possible. Knowing the location of these vessels may allow surgeons to avoid vascular damage and improve regeneration of injured/repaired nerves. As imaging technology continues to evolve, novel methods have emerged to create detailed images of vasculature in vivo. 1 However, the literature is lacking in data specifically assessing the feeding and draining vessels of nerves. In this review, we highlight the significance, historical methods, and emerging techniques for imaging the arterial supply of peripheral nerves.

## Nerve Function and Blood Supply

Early reports detailing the interplay between nerve injury and vascular supply were presented by Rydevik et al, Rydevik and Lundborg, and Lundborg in the 1970s. 2 3 4 These articles evaluated structure, function, and response to injury, as well as the extrinsic and intrinsic vasculature of nerves. More recently, researchers have investigated the effect of nerve compression on intraneural blood flow and blood–nerve barrier permeability. 5 Peripheral nerve dysfunction and repair continue to present challenges despite advances in both imaging and treatment modalities. While functional testing, such as electromyography (EMG) and nerve conduction studies (NCS), may tell us whether or not the nerve is communicating, they do little to address the cause of diminished nerve function.

One possible explanation for persistent nervous deficiencies is a lack of vascular perfusion. It is well documented that angiogenesis is intimately connected with reinnervation, arborization, and growth of peripheral nerves. 5 For example, it has been shown in a model of transplanted human skin equivalents that vascularization precedes the process of innervation, indicating that nervous tissue proliferation is driven by nutritional and trophic factors provided by the vascular system. 6 While some have argued that an intraneural vascularization happens concurrently with the proliferation of fascicles, little is known about the specific role of revascularization in nerve regeneration. 7 8

Given that inflammatory changes, axon growth, and cell regeneration require a large number of nutritive substances and oxygen, it is not surprising that damaged microcirculation can hamper neural regeneration and functional recovery. 5 After peripheral nerve damage, the diameter of blood vessels of the intraneural vascular (INV) system increases due to the effects of neuropeptides such as substance P (SP) and calcitonin gene–related peptide (CGRP). Vasodilatation, associated with the release of monocyte chemoattractant protein-1 (MCP-1) by Schwann's cells (SC), allows macrophage recruitment. 9 Migrating and resident macrophages then secrete VEGF. This growth factor in turn stimulates neoangiogenesis, supplying the oxygen and nutrients needed to form the “bands of Büngner” by SCs. The molecular interactions between SCs, macrophages, and endothelial cells of neovessels have not yet been elucidated. 9

Given the apparently integral role of vascularization in the maintenance and regeneration of nervous tissue, physicians and scientists have attempted to identify the vascular connections that feed peripheral nerves. In what follows, historical methods of observation will be evaluated, as well as current and emerging imaging techniques that may be of use in visualizing these structures.

## Cadaveric Mapping of the Blood Supply to Peripheral Nerves

Few cadaveric studies have been performed to map the connections of peripheral arteries to the vasa nervorum. 10 11 12 13 These had limitations inherent in all cadaveric studies, including possible loss of in vivo characteristics through the storage and preparation process, inability to account for anatomical variation, and variability of injection pressure and/or dissection technique which may have influenced which vessels did or did not stain. 12 While they are not without limitations, these studies implicate the potential value of mapping the arterial supply of peripheral nerves. One study of 15 forearms found that a constant branch to the median nerve arises from the radial artery approximately 5-cm proximal to the radial styloid process. This suggests that it may be possible to advance the median nerve at the wrist, while retaining vascular input at a common site of nerve repair. 13 Another study identified the potential for improvements in free vascular nerve grafting. 11 12 Others indicated that knowing the location of these vessels could lead to superior blood supply for neurovascular and neurocutaneous flaps. 10 11

## Historical Imaging of Vascular Structures

Characterization of vascular structures has historically been done using techniques such as: digital subtraction angiography (DSA), ultrasound (US), magnetic resonance imaging (MRI), and laser angiography. DSA provides high temporal (≤1 second frame time) and high spatial resolution (≤1 mm) but is an invasive procedure that uses iodinated contrast material and radiation to acquire projected images. US was later used as a noninvasive method of visualizing blood flow to nerves but has limitations including inability to penetrate tissue, small imaging field, and highly operator-dependent examination quality. An MRI protocol using T1- and T2-weighted imaging is currently the preferred method as it is able to characterize the nervous structures and surrounding tissue noninvasively with high spatial resolution. A newer subset of MRI protocols termed MR angiography (MRA) has shown utility in evaluating vascular structures in vivo. 14 Optical coherence tomography (OCT) was later developed to provide high-resolution images of superficial blood flow. 15 Recently, laser angiography has combined some advantages of US and MR techniques to provide rapid imaging of tissue perfusion intraoperatively. 16

## Ultrasound/Doppler Angiography

Ultrasonography is a low-cost tool that has shown utility in imaging neuro vasculature. In recent years, the spatial resolution of US has improved dramatically, and its applications have evolved to include imaging of the peripheral nervous system. 17 In 2010, a study was performed on New Zealand white rabbits in an effort to quantitatively assess peripheral nerve blood perfusion using contrast-enhanced US (CEUS). Using CEUS, the supplying arteries to the femoral nerves were identified, suggesting that this methodology could be useful for visualizing nerve perfusion. 18

One study of human subjects attempted to observe the collateral vessels in the knee in patients with Buerger's disease using simple Doppler US. Researchers were able to identify collateral vessels, originating from vasa nervorum of the tibial nerve in instances of popliteal artery occlusion. 19 Results of CEUS and Doppler US studies suggest that feeding vessels of peripheral nerves can be adequately visualized in vivo.

One disadvantage of US techniques is the need for a skilled operator which can have dramatic effects on image quality and precision. 17 Other disadvantages include depth of visual field and occurrence of artifact from surrounding structures. 17

## Magnetic Resonance Imaging and Magnetic Resonance Neurography

MR neurography (MRN) protocols have been developed to provide insights into the fascicular structure of nerves. While this has been useful in evaluating nerve injuries, fascicular structure is not necessarily determinative of functional outcome. 20 The specificities of MR signal changes seen in histologically controlled studies are still a contentious matter, as possible causes of signal alteration may include regeneration, degeneration in the distal nerve, or any combination of the two. 21 Potential factors complicating analysis include high myelin turnover, presence of inflammatory mediators, changes in the blood–nerve barrier, axoplasmic flow caused by axon and nerve sheath degeneration, and increased water content in an enlarged endoneurial space. 21 22 23

Though the literature is limited, MRI has been used to map vascular supply to peripheral nerves. One study using 3-T MRI with fat-suppressed, contrast-enhanced T1 images was able to identify intraneural vessels in 26% of patients, and even identified some perforating vessels along the sciatic nerve. 24 Unfortunately, the images in this study had relatively low resolution and likely missed many smaller vascular elements.

While 7-T MRI improves resolution, it still does not clearly delineate the nerves and vessels in close proximity. 25 The 7-T scanners provide better resolution than 3-T scanners due to their increased signal-to-noise ratio (SNR). However, the radiofrequency (RF) field inhomogeneity corrections are more difficult to execute in 7-T than in 3-T MRI. Image quality is often improved in ultrahigh-resolution imaging by using more coil elements (or receiver channels). This increases the parallel imaging acceleration factor to reduce imaging time. 26 While MRI and MRN technologies are continually improving, current techniques are not suitable for clinical use to visualize nervous vasculature.

## Magnetic Resonance Perfusion

There are two MR perfusion methods currently in use: dynamic susceptibility contrast MRI (DSC-MRI) and arterial spin labeling (ASL). DSC requires injection of a contrast agent and subsequent acquisition of a fast time series of images that track the contrast through vascular structures. ASL applies a spatially selective RF excitation pulse to a region containing large arteries and allowing this “labeled” blood to flow into the imaging plane. As T1 decay of the label takes place, it is followed by subtraction of labeled images from unlabeled control images, producing a measured signal that is proportional to blood flow. Typically, multiple images must be averaged to achieve a low SNR. 23

MR perfusion is commonly used to study cerebrovascular disorders including: acute stroke, subacute and chronic ischemia, stenotic-occlusive disease, moyamoya disease, and vasospasm secondary to subarachnoid hemorrhage. MR perfusion can assess collateral flow, duration of occlusion and hypoperfusion, and presence and/or extent of recanalization.

One early study used MR perfusion to evaluate median nerve circulation. However, their analysis was mostly qualitative, enhanced (high signal) nerves were classified as edemic and unenhanced (reduced signal) nerves as ischemic. 27 Soon after, a study quantifying optic nerve blood flow in rats was performed using Gadolinium-diethylenetriamine pentaacetic acid contrast with T2-weighted MRI. 28 Subsequent calculations used signal intensity values to determine mean basal blood flow in the anterior and posterior portions of the optic nerve. 28

Recently, Bäumer et al used dynamic contrast-enhanced MR perfusion to evaluate human peripheral nerve perfusion in 102 patients (43 control and 59 neuropathy). 29 Researchers measured signal intensity on multiple sequences at weights of T2 and T1 to determine the arterial input function and mean contrast uptake. Using the Patlak model, the authors then calculated nerve–blood volume (NBV) and blood–nerve permeability (K-trans). 29

While MR perfusion is advantageous for generalized perfusion of a local area, other techniques, such as MRA, are often used as a baseline to further categorize results. 30 MR perfusion can be used to identify nerve vasculature; however, other methods offer a more direct approach.

## Magnetic Resonance Angiography

### Time-of-Flight Magnetic Resonance Angiography

Time-of-flight (TOF) techniques distinguish between flowing blood and stationary tissues by manipulating the magnitude of the magnetization, such that the magnitude of the magnetization from the moving spins is large and the magnetization from the stationary spins is small. This creates a large signal from moving blood spins and a small signal from stationary tissue spins. 31

TOF-MRA has been used clinically to visualize the hemodynamics of intracranial arteries, particularly in patients suspected of having intracranial aneurisms. 32 33 34 Upon the development of phase-contrast MRA (PC-MRA), three-dimensional (3D) TOF-MRA was shown to be an inferior diagnostic tool. 33

More recently, Shibukawa et al developed a novel time-resolved MRA called four-dimensional (4D) TOF-MRA using saturation pulse. 32 Another TOF-MRA protocol was developed using 7-T imaging in combination with reduced specific absorption rate and venous suppression. 35 While both newer methods demonstrated superiority compared with 3D TOF-MRA, a direct comparison was not performed for PC-MRA or contrast-enhanced MRA (CE-MRA).

TOF-MRA shows some benefit in mapping vasculature due to its sensitivity to blood flow and direction. Unfortunately, TOF imaging requires a long acquisition times and images are often obscured by artifacts. 36 These properties make TOF-MRA an unfavorable imaging option for nervous vascular structures.

### Phase-Contrast Magnetic Resonance Angiography

Phase-contrast (PC) techniques distinguish between flowing blood and stationary tissues by manipulating the phase of the magnetization, such that the phase of the magnetization of the stationary spins is 0 and the phase of moving spins is non-0. In phase difference images, faster moving spins produce a larger signal, and spins moving in one direction are assigned a bright (white) signal, whereas spins moving in the opposite direction are assigned a dark (black) signal. Thus, the vascular anatomy can be visualized, as well as the speed and direction of the blood flow. 31

Recent advances in PC-MRA acquisition and reconstruction have allowed for high-resolution images to be obtained with shorter imaging times. Nearly all variations of PC-MRA utilize parallel imaging directly with techniques, such as SENSE or GRAPPA, but also with indirect methods such as localized coils. In the case of Cartesian (parallel) imaging, scan time is reduced by a factor of 2 to 4. Unfortunately, this still fails to adequately reduce scan times for clinically relevant coverage and spatial resolution (<1 mm). 37

Another method for reducing scan times utilizes non-Cartesian sampling trajectories which either collect more data per excitation (spiral trajectories) or allow greater under sampling of the imaging volume without obscuring artifacts (radial trajectories). Non-Cartesian imaging also reduces echo time, improving imaging of complex flow, and producing higher spatial resolution. 38 39 40 A third time-reduction method uses a reconstruction based on the fact that the majority of signal is from nonmoving tissues and consequently the same in all time frames. This method accelerates acquisition time, but requires substantial computational power. 37

In 2004, Bilecen and colleagues 41 introduced a novel MRA technique that combines subsystolic arm pressure with multiphasic continuous acquisition of a spoiled 3D gradient echo sequence. This technique requires only 24 seconds to record one dataset. Unfortunately, it provides no information on hemodynamics. 42 Winterer et al addressed this issue by designing a protocol using ultra rapid time-resolved 3D imaging, with accelerated acquisition employing interleaved Stochastic Trajectories (TWIST) and generalized autocalibrating partially parallel acquisition (GRAPPA) in 3-T CE-MRA. Using this protocol, they demonstrated that time-resolved CE-MRA is four times faster than PC-MRA and reduces venous contamination in imaging hand arteries. 42 A recent article described the use of a minimum phase Shinnar-Le Roux excitation and the “time‐optimal variable‐rate selective excitation” method to shorten radio frequency (RF) and slab‐select waveforms, further reducing the time needed to acquire images. 43

In a clinical setting, PC-MRA can show vascular morphology and gives quantitative measurements of blood velocity. Velocity data allows observers to derive hemodynamic parameters such as flow volume, relative wall shear stress, streamlines, vorticity, and pressure gradients. PC-MRA has combined anatomic vessel wall imaging, lumen visualization, and physiologic data to augment the characterization of intracranial arterial stenosis, aneurysms, vascular malformations, and dural sinus pathology. 37 Not all of these benefits are needed for nerve-specific applications. While PC-MRA has demonstrated capability for visualizing small vasculature, many hospitals may lack the resources to implement improved protocols.

### Contrast-Enhanced Magnetic Resonance Angiography

CE techniques distinguish between blood and stationary tissues by manipulating the magnitude of the magnetization (i.e., TOF techniques) and by intravenously injecting a contrast agent into the vascular system to selectively shorten the T1 of the blood. By implementing a T1-weighted imaging sequence during the first pass of the contrast agent, images can be produced that show arteries with striking contrast relative to surrounding stationary tissues and veins. 31

CE-MRA was introduced for clinical use approximately 25 years ago, with early iterations providing 3- to 4-mm spatial resolution in 30 seconds of acquisition timeframes. Since then, improvements in spatial resolution and acquisition time have allowed high-resolution, 3D time-resolved studies. 44 These newer CE-MRA techniques have been utilized to provide structural details of vasculature and flowmetry data in peripheral feeding and draining vessels of the forearm, hand, thigh, and foot. This data helped to progress our understanding of vascular malformation. 14

In 2007, CE-MRA was used to determine the level and side of the suspected spinal dural arteriovenous fistula (SDAVF) and the feeding arteries in spinal arteriovenous malformations (SAVMs). These findings were corroborated via comparison with DSA of the same tissue. CE-MRA was able to reliably detect or exclude various types of spinal AV abnormalities and localize the predominant arterial feeder of most spinal AV shunts. These findings indicate that MRA may serve as a noninvasive means of focusing subsequent DSA in these patients. 45

While CE-MRA requires an intravenous contrast to be administered, there are significant benefits to this technique in mapping perfusion of peripheral nerves. High temporal and spatial resolution can be gained from accurate timing of the contrast agent bolus, as well as the low likelihood of flow-related artifacts. 36 Given the low acquisition times of newer protocols, CE-MRA is a promising method of visualizing nerve perfusion in a clinical setting.

## Optical Coherence Tomography

OCT is a relatively new imaging technique that uses low coherence light to produce a 2D or 3D image. OCT is an ideal candidate for obtaining a submicrometer resolution, and is commonly used medically to obtain high-resolution images of the retina. 15

A study performed on rats in vivo using OCT aimed to detect cerebral edema status post cerebral artery occlusion, and was able to detect gradual ischemic changes with high accuracy, similar to the results of an MRI. 46 Another study compared five different systems of CTA angiography and how successfully they can evaluate optical vasculature. All systems trialed were able to obtain accurate depictions of vasculature but motion artifacts and acquisition times posed problems. 15

These studies demonstrate that OCT appears reliable for imaging of superficial structures, but few studies have attempted to visualize deeper structures. One such study attempted to evaluate coronary arteries following stent implantation. A high amount of resolution was achieved, but there were still shortcomings of imaging deep vessels. The study mentions that OCT needs complete blood clearance from the lumen and that thrombi casts large shadows of artifact. 47 Even when deeper structures are visualized, they are often compared with MRI and MRA for validity, suggesting that other methods for mapping feeding vessels may be more reliable. 48

## Optical Microangiography

Optical microangiography (OMAG) is a subset of the OCT technique designed specifically for imaging blood flow in microcirculation. It is based on the principal that moving blood scatters light in such a way that it can be captured and adjusted into a 3D image. 49

This technique is frequently applied to visualizing cutaneous blood flow. OMAG is a reliable biomarker in many pathological situations such as lesions and basal cell carcinoma. 50 In vivo experiments on mice have been performed which demonstrate a noninvasive technique of visualizing cerebral blood flow. The 3D rendering from the experiments showed that vessels as small as 15 μm can be observed. 49 51

None of these studies have evaluated OMAG in the context of peripheral nerve vasculature; however, the ability to visualize microcirculation could be beneficial. Most studies using OMAG, however, view microcirculation at relatively superficial levels. 50 Few studies have been performed which would indicate deeper tissues can be visualized in a similar manner to cutaneous vasculature. 46 47 48 These limitations may impact accuracy of observing deep feeder vessels.

## Laser Angiography

Laser angiography techniques have recently been developed to combine the real-time acquisition of Doppler angiography and precision of MRA techniques, without the highly operator-dependent image quality (US) or long acquisition times (MRA). 52 Two common devices are the SPY Intraoperative Perfusion Assessment System (Novadaq Technologies Inc., Richmond, BC, Canada) and SPY Elite Fluorescence imaging system (Stryker Inc., Kalamazoo, Michigan, United States), both of which utilize indocyanine green (ICG), a fluorescent agent that binds to plasma proteins with a half-life of 3 to 5 minutes. 52 53 The pharmacokinetic properties of ICG allow for rapid clearance and repeated intraoperative visualization of tissue perforation.

Laser angiography has been used successfully to monitor tissue perfusion in a variety of settings, including colorectal, plastic, endocrine, ophthalmologic, and vascular surgery. 54 While the majority of literature is focused on tissue resection and/or flap design, one case series described visualization of the sciatic nerve perivascular system in three pediatric sarcoma patients during limb salvage surgery. 16 While depth of structures limit its application in mapping common arterial inputs and/or venous drainage, laser angiography may be clinically useful in visualizing these vessels intraoperatively.

## Discussion

MRI and MRN provide ideal imaging of fascicular structure of nerves. However, fascicular regeneration does not always correlate with return to function. 20 As such, additional techniques are needed to fully assess the spectrum of factors such as blood perfusion that are known to affect recovery. There are substantial difficulties associated with imaging the vessels that supply peripheral nerves. The vessels are small and may be located in deep tissue layers depending on the nerve in question. For example, vessels feeding the femoral nerve are surrounded by several dense and vascular structures that increase susceptibility to artifact. 18

In addition to inherent challenges of size and tissue depth, random variation can be observed in human vascular networks, such as accessory or aberrant arterial supply. 55 While arterial variation may not be exceedingly common, it is a variable that is difficult to control for, especially in current cadaveric models where sample sizes are necessarily small. Given the potential anatomic variation in individuals, noninvasive methods are essential for mapping peripheral vasculature.

Historical imaging techniques used in nervous and vascular tissue were often unable to adequately show vascular structures at this depth with limitations including invasive nature of the procedure, poor resolution, inability to show microscopic structures, or the need for highly skilled operators. 17 26 36 37 Recent developments have provided improved resolution, less-invasive imaging, and devices that are less prone to operator error. Emerging techniques may be divided by their respective contributions to nerve treatment algorithms.

US/Doppler, OCT, and laser angiography are most useful in a clinical setting to visualize superficial structures in individual patients. US is currently limited by depth of structures and necessary operator skill. Despite these shortcomings, US is beneficial in intraoperative settings due to its mobility and rapid function. CEUS has been used successfully to measure intraneural vascularity in a variety of nerve pathologies, most commonly compressive neuropathy. 56 OCT and OMAG excel at visualizing morphology of microcirculation but may also be limited by depth of structures. 49 50 51 Laser angiography provides fast, accurate imaging with minimal required skill and can be used intraoperatively to identify and preserve blood flow to nerves. 16

Various MR techniques are most useful for mapping common vessels to develop a knowledge base for cases in which direct visualization is untenable. These may also be beneficial for nonacute visualization in individual cases, either pre- or postoperatively. While the MR perfusion has been used to detect intraneural edemic/ischemic conditions in peripheral nerves, this is an indirect method of determining the location of feeder vessels. 27 TOF-MRA and PC-MRA provide high-resolution images by utilizing the contrast between flowing blood and stationary tissue. 37 Studies assessing peripheral vascular disease indicate that these may adequately image neurovascular structures. 57 Given the long acquisition times associated with TOF and PC-MRA, 37 they will likely be of greater use in studies mapping common feeder vessels than in acute or subacute clinical cases.

CE-MRA produces high-resolution images of deep structures with little to no artifact and reduced acquisition time compared with TOF or PC-MRA. 36 Studies have demonstrated high accuracy of CE-MRA in visualizing feeding and draining vessels with excellent contrast between arteries and surrounding tissue. 14 31 Given its success as a noninvasive alternative to DSA in patients with vascular malformations, CE-MRA may be a valuable tool for mapping nerve vasculature and nonacute visualization in individual patients. 44

Currently, US imaging has demonstrated the greatest clinical utility. When a skilled operator is available, US allows surgeons to evaluate nervous vascularization to corroborate clinical symptoms and direct treatment. Multiple studies have shown that US has improved diagnostic accuracy compared with MRI. 58 59 Future diagnostic and/or monitoring algorithms should incorporate both ultrasonography and CE-MRA to address the broad spectrum of patient dynamics and available hospital resources.

## Conclusion

While imaging technologies are rapidly advancing, studies evaluating their efficacy in the context peripheral nerve vasculature are limited. Accurate imaging and mapping of these vessels may augment surgeons' ability to perform repairs without compromising nerve perfusion, ultimately resulting in improved postoperative outcomes. The published data, while limited, support further investigation to determine which modality(s) might be best suited to the task of locating arterial inflow to peripheral nerves. Whether by direct visualization in individual cases or by mapping structures conserved across patients, the techniques presented in this review may translate to improve patient outcomes following nerve injury.
